# 4-Formyl­phenyl 2,3,4,6-tetra-*O*-acetyl-β-d-glucopyran­oside

**DOI:** 10.1107/S1600536811008099

**Published:** 2011-03-09

**Authors:** Thorsten Heidelberg, Rusnah Syahila Duali Hussen, Nasrul Zamani Mohd Rodzi, Seik Weng Ng, Edward R. T. Tiekink

**Affiliations:** a Department of Chemistry, University of Malaya, 50603 Kuala Lumpur, Malaysia

## Abstract

The pyran­oside ring in the title compound, C_21_H_24_O_11_, has a chair conformation with the substituted benzene ring occupying an equatorial position. The crystal packing is dominated by C—H⋯O inter­actions that lead to the formation of supra­molecular layers in the *ab* plane.

## Related literature

For synthesis, see: Bao *et al.* (2004 ▶); Hongu *et al.* (1999 ▶); Patil & Ravindranathan Kartha (2008 ▶). For the natural anti-oxidant glucosyl­ated resveratrol, see: La Torre *et al.* (2004 ▶). For the biological activity of related structures, see: Wen *et al.* (2008 ▶); Yan *et al.* (2009 ▶). For the structure of the isomeric allopyran­oside and galactose derivatives, see: Ye *et al.* (2009 ▶); Hussen *et al.* (2011 ▶). For conformational analysis, see: Cremer & Pople (1975 ▶).
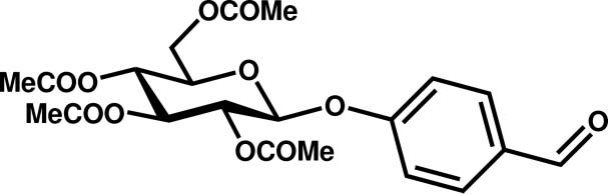


## Experimental

### 

#### Crystal data

C_21_H_24_O_11_
*M* *_r_* = 452.40
Triclinic, 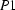

*a* = 5.7868 (2) Å
*b* = 8.9166 (3) Å
*c* = 11.4716 (3) Å
α = 102.473 (3)°
β = 93.481 (2)°
γ = 102.780 (3)°
*V* = 559.96 (3) Å^3^
*Z* = 1
Cu *K*α radiation
μ = 0.94 mm^−1^
*T* = 100 K
0.30 × 0.30 × 0.20 mm

#### Data collection

Agilent Supernova Dual diffractometer with an Atlas detector
Absorption correction: multi-scan (*CrysAlis PRO*; Agilent Technologies, 2010 ▶) *T*_min_ = 0.919, *T*_max_ = 1.000
7392 measured reflections
4097 independent reflections
4087 reflections with *I* > 2σ(*I*)
*R*_int_ = 0.021

#### Refinement

*R*[*F* ^2^ > 2σ(*F* ^2^)] = 0.041
*wR*(*F* ^2^) = 0.115
*S* = 1.07
4097 reflections
293 parameters
3 restraints
H-atom parameters constrained
Δρ_max_ = 0.27 e Å^−3^
Δρ_min_ = −0.17 e Å^−3^
Absolute structure: Flack (1983 ▶), 1855 Friedel pairs
Flack parameter: -0.02(12)

### 

Data collection: *CrysAlis PRO* (Agilent Technologies, 2010 ▶); cell refinement: *CrysAlis PRO*; data reduction: *CrysAlis PRO*; program(s) used to solve structure: *SHELXS97* (Sheldrick, 2008 ▶); program(s) used to refine structure: *SHELXL97* (Sheldrick, 2008 ▶); molecular graphics: *ORTEP-3* (Farrugia, 1997 ▶) and *DIAMOND* (Brandenburg, 2006 ▶); software used to prepare material for publication: *publCIF* (Westrip, 2010 ▶).

## Supplementary Material

Crystal structure: contains datablocks global, I. DOI: 10.1107/S1600536811008099/ez2235sup1.cif

Structure factors: contains datablocks I. DOI: 10.1107/S1600536811008099/ez2235Isup2.hkl

Additional supplementary materials:  crystallographic information; 3D view; checkCIF report

## Figures and Tables

**Table 1:** Hydrogen-bond geometry (Å, °)

*D*—H⋯*A*	*D*—H	H⋯*A*	*D*⋯*A*	*D*—H⋯*A*
C1—H1⋯O5^i^	1.00	2.51	3.356 (2)	143
C3—H3⋯O5^i^	1.00	2.35	3.207 (2)	143
C6—H6*A*⋯O9^ii^	0.99	2.40	3.324 (2)	155
C8—H8*C*⋯O11^iii^	0.98	2.54	3.475 (3)	160

Symmetry codes: (i) 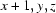; (ii) 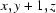; (iii) 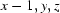.

**Fig. 1.**
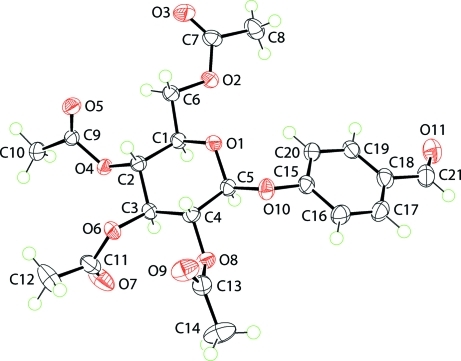
Molecular structure, showing the atom-labelling scheme and displacement ellipsoids at the 70% probability level.

**Fig. 2.**
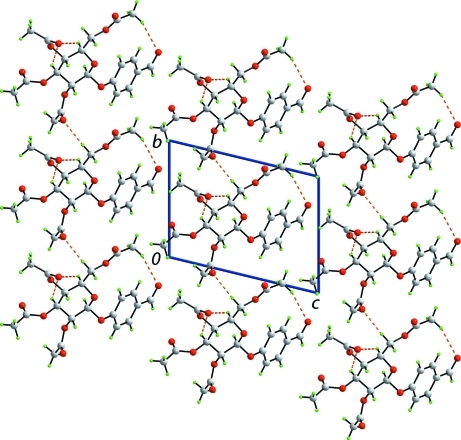
A view in projection down the a axis of the unit-cell contents highlighting the stacking of layers. The C—H···O interactions are shown as orange dashed lines.

## supplementary crystallographic information

### Comment

The title compound, 4-formyl-phenyl 2,3,4,6-tetra-*O*-acetyl-β-*D*-glucopyranoside, a known species (Bao *et al.*, 2004, Hongu *et al.*, 1999; Patil *et al.*; 2008), which has been used for the preparation of potential pharmaceutically active compounds (Wen *et al.*, 2008; Yan *et al.*, 2009) was prepared as a precursor for the synthesis of glucosylated resveratrol, an interesting natural antioxidant (La Torre *et al.*, 2004). The present analysis complements the recent report of the isomeric galactose derivative, see: Hussen *et al.* (2011).

The structure determination, Fig. 1, confirms the relative stereochemistry as well as the absolute structure, *i.e. R*, *R*, *S*, *R* and *S* for C1–C5, respectively. The pyranoside ring has a chair conformation as seen in the puckering parameters (Cremer & Pople, 1975): puckering amplitude (Q) = 0.6016 (18) Å, θ = 172.53 (16) °, and φ = 178.0 (14) °. Around the ring, all substituents are equatorial.

The crystal packing is dominated by C–H···O interactions, Table 1, involving carbonyl atoms as acceptors and methine-, methylene methyl-H as the donors. The carbonyl-O5 atom is bifurcated, spanning two methine-H atoms of a neighbouring molecule to form a supramolecular chain along the *a* axis. Altogether, the C–H···O interactions lead to the formation of supramolecular layers that stack along the *c* axis, Fig. 2.

The present report complements the structures reported recently for the isomeric allopyranoside (Ye *et al.*, 2009) and galactose (Duali Hussen *et al.*, 2011) derivatives.

### Experimental

2,3,4,6-Tetra-*O*-acetyl-α-*D*-gluctopyranosyl bromide (4.0 g) and 4-hydroxybenzaldehyde (3.0 g) were dissolved in chloroform (30 ml) and the mixture treated with a solution of aqueous solution (15 ml) of sodium carbonate (2.7 g) and tetrabutylammonium bromide (0.7 g). The mixture was heated to reflux under vigorous stirring overnight, after which ethyl acetate was added and the organic layer was washed three times with sodium hydroxide solution (1 N) to remove remaining phenols. After drying the solution over magnesium sulfate and evaporation of the solvent, the target product (2.0 g, 45%) was obtained by crystallization from ethanol. Better crystals were obtained from 2-propanol.

^1^H NMR (400 MHz, CDCl_3_): δ 9.92 (s; CHO), 7.85 & 7.09 (AB syst; aromatic 4H), 5.34–5.26 & 5.24–5.14 (2 m, 2 *x* 2H; H1–H4), 4.27 (dd; H6a), 4.16 (dd; H6b), 3.92 (ddd; H5), 2.05–2.03 (3 s, 12H; Ac); ^3^J_4,5_ = 10.0 Hz, ^3^J_5,6a_ = 5.0 Hz, ^3^J_5,6 b_ = 2.5 Hz and ^2^J_6_ = 12.0 Hz.

### Refinement

Carbon-bound H-atoms were placed in calculated positions (C—H 0.95 to 1.00 Å) and were included in the refinement in the riding model approximation, with *U*_iso_(H) set to 1.2 to 1.5*U*_equiv_(C).

### Crystal data

**Table:**

C_21_H_24_O_11_	*Z* = 1
*M**_r_* = 452.40	*F*(000) = 238
Triclinic, *P*1	*D*_x_ = 1.342 Mg m^−^^3^
Hall symbol: P 1	Cu *K*α radiation, λ = 1.54184 Å
*a* = 5.7868 (2) Å	Cell parameters from 7285 reflections
*b* = 8.9166 (3) Å	θ = 4.0–74.1°
*c* = 11.4716 (3) Å	µ = 0.94 mm^−^^1^
α = 102.473 (3)°	*T* = 100 K
β = 93.481 (2)°	Block, colourless
γ = 102.780 (3)°	0.30 × 0.30 × 0.20 mm
*V* = 559.96 (3) Å^3^	

### Data collection

**Table:**

Agilent Supernova Dual diffractometer with an Atlas detector	4097 independent reflections
Radiation source: SuperNova (Cu) X-ray Source	4087 reflections with *I* > 2σ(*I*)
Mirror	*R*_int_ = 0.021
Detector resolution: 10.4041 pixels mm^-1^	θ_max_ = 74.3°, θ_min_ = 4.0°
ω scans	*h* = −7→7
Absorption correction: multi-scan (*CrysAlis PRO*; Agilent Technologies, 2010)	*k* = −9→10
*T*_min_ = 0.919, *T*_max_ = 1.000	*l* = −13→14
7392 measured reflections	

### Refinement

**Table:**

Refinement on *F*^2^	Secondary atom site location: difference Fourier map
Least-squares matrix: full	Hydrogen site location: inferred from neighbouring sites
*R*[*F*^2^ > 2σ(*F*^2^)] = 0.041	H-atom parameters constrained
*wR*(*F*^2^) = 0.115	*w* = 1/[σ^2^(*F*_o_^2^) + (0.0908*P*)^2^ + 0.072*P*] where *P* = (*F*_o_^2^ + 2*F*_c_^2^)/3
*S* = 1.07	(Δ/σ)_max_ = 0.001
4097 reflections	Δρ_max_ = 0.27 e Å^−^^3^
293 parameters	Δρ_min_ = −0.17 e Å^−^^3^
3 restraints	Absolute structure: Flack (1983), 1855 Friedel pairs
Primary atom site location: structure-invariant direct methods	

### Special details

**Table:**

Geometry. All e.s.d.'s (except the e.s.d. in the dihedral angle between two l.s. planes) are estimated using the full covariance matrix. The cell e.s.d.'s are taken into account individually in the estimation of e.s.d.'s in distances, angles and torsion angles; correlations between e.s.d.'s in cell parameters are only used when they are defined by crystal symmetry. An approximate (isotropic) treatment of cell e.s.d.'s is used for estimating e.s.d.'s involving l.s. planes.
Refinement. Refinement of *F*^2^ against ALL reflections. The weighted *R*-factor *wR* and goodness of fit *S* are based on *F*^2^, conventional *R*-factors *R* are based on *F*, with *F* set to zero for negative *F*^2^. The threshold expression of *F*^2^ > σ(*F*^2^) is used only for calculating *R*-factors(gt) *etc*. and is not relevant to the choice of reflections for refinement. *R*-factors based on *F*^2^ are statistically about twice as large as those based on *F*, and *R*- factors based on ALL data will be even larger.

### Fractional atomic coordinates and isotropic or equivalent isotropic displacement parameters (Å^2^)

**Table:**

	*x*	*y*	*z*	*U*_iso_*/*U*_eq_	
O1	0.9987 (2)	0.49931 (15)	0.49910 (11)	0.0174 (3)	
O2	1.0120 (2)	0.81529 (16)	0.57609 (11)	0.0218 (3)	
O3	0.7432 (3)	0.95346 (19)	0.63881 (14)	0.0305 (3)	
O4	0.7892 (2)	0.55996 (15)	0.20730 (10)	0.0167 (3)	
O5	0.4356 (2)	0.59974 (16)	0.26001 (12)	0.0208 (3)	
O6	0.7723 (2)	0.23423 (15)	0.15960 (11)	0.0192 (3)	
O7	1.0136 (3)	0.2794 (2)	0.01679 (14)	0.0385 (4)	
O8	1.1640 (2)	0.17486 (15)	0.30105 (11)	0.0196 (3)	
O9	0.8768 (3)	−0.04604 (17)	0.28892 (15)	0.0297 (3)	
O10	1.1933 (2)	0.31718 (15)	0.54454 (11)	0.0204 (3)	
O11	2.0808 (3)	0.73176 (19)	0.92751 (14)	0.0330 (4)	
C1	0.9617 (3)	0.5930 (2)	0.41525 (15)	0.0166 (3)	
H1	1.1190	0.6460	0.3937	0.020*	
C2	0.8116 (3)	0.4800 (2)	0.30264 (15)	0.0155 (3)	
H2	0.6503	0.4316	0.3225	0.019*	
C3	0.9368 (3)	0.3515 (2)	0.25138 (15)	0.0163 (3)	
H3	1.0817	0.3980	0.2159	0.020*	
C4	1.0071 (3)	0.2710 (2)	0.34749 (16)	0.0171 (3)	
H4	0.8624	0.2051	0.3712	0.021*	
C5	1.1452 (3)	0.3968 (2)	0.45629 (15)	0.0168 (3)	
H5	1.2968	0.4572	0.4343	0.020*	
C6	0.8401 (3)	0.7168 (2)	0.47781 (16)	0.0187 (3)	
H6A	0.7979	0.7799	0.4222	0.022*	
H6B	0.6930	0.6665	0.5080	0.022*	
C7	0.9388 (3)	0.9279 (2)	0.65168 (16)	0.0220 (4)	
C8	1.1278 (4)	1.0105 (3)	0.75474 (18)	0.0291 (4)	
H8A	1.1138	1.1193	0.7845	0.044*	
H8B	1.2855	1.0119	0.7277	0.044*	
H8C	1.1077	0.9543	0.8194	0.044*	
C9	0.5824 (3)	0.6053 (2)	0.19069 (15)	0.0163 (3)	
C10	0.5674 (3)	0.6549 (3)	0.07482 (17)	0.0234 (4)	
H10A	0.4287	0.7001	0.0685	0.035*	
H10B	0.5507	0.5627	0.0077	0.035*	
H10C	0.7128	0.7344	0.0721	0.035*	
C11	0.8311 (4)	0.2113 (2)	0.04513 (17)	0.0250 (4)	
C12	0.6319 (5)	0.0922 (3)	−0.0371 (2)	0.0428 (6)	
H12A	0.6747	0.0749	−0.1195	0.064*	
H12B	0.4866	0.1315	−0.0340	0.064*	
H12C	0.6041	−0.0078	−0.0117	0.064*	
C13	1.0775 (3)	0.0158 (2)	0.27726 (16)	0.0214 (4)	
C14	1.2625 (4)	−0.0679 (3)	0.2328 (3)	0.0385 (5)	
H14A	1.1877	−0.1802	0.1990	0.058*	
H14B	1.3833	−0.0573	0.2997	0.058*	
H14C	1.3381	−0.0212	0.1706	0.058*	
C15	1.3937 (3)	0.3914 (2)	0.62530 (15)	0.0185 (4)	
C16	1.5446 (4)	0.2974 (3)	0.64956 (18)	0.0230 (4)	
H16	1.5095	0.1883	0.6104	0.028*	
C17	1.7455 (4)	0.3644 (3)	0.73110 (18)	0.0249 (4)	
H17	1.8490	0.3008	0.7482	0.030*	
C18	1.7984 (3)	0.5252 (2)	0.78886 (16)	0.0229 (4)	
C19	1.6452 (3)	0.6178 (2)	0.76354 (15)	0.0216 (4)	
H19	1.6803	0.7268	0.8028	0.026*	
C20	1.4427 (3)	0.5528 (2)	0.68196 (16)	0.0209 (4)	
H20	1.3390	0.6162	0.6647	0.025*	
C21	2.0148 (4)	0.5937 (3)	0.87497 (18)	0.0279 (4)	
H21	2.1099	0.5244	0.8905	0.034*	

### Atomic displacement parameters (Å^2^)

**Table:**

	*U*^11^	*U*^22^	*U*^33^	*U*^12^	*U*^13^	*U*^23^
O1	0.0207 (6)	0.0178 (6)	0.0153 (5)	0.0077 (5)	0.0026 (4)	0.0040 (5)
O2	0.0222 (6)	0.0195 (7)	0.0197 (6)	0.0055 (5)	−0.0015 (5)	−0.0030 (5)
O3	0.0332 (8)	0.0268 (8)	0.0305 (7)	0.0149 (6)	0.0023 (6)	−0.0032 (6)
O4	0.0163 (6)	0.0200 (6)	0.0160 (5)	0.0071 (4)	0.0020 (4)	0.0061 (4)
O5	0.0175 (6)	0.0221 (7)	0.0231 (6)	0.0063 (5)	0.0035 (5)	0.0043 (5)
O6	0.0202 (6)	0.0176 (6)	0.0160 (6)	0.0022 (5)	−0.0017 (5)	−0.0006 (5)
O7	0.0419 (9)	0.0422 (9)	0.0211 (7)	−0.0038 (7)	0.0091 (6)	−0.0018 (6)
O8	0.0178 (6)	0.0166 (6)	0.0245 (6)	0.0057 (5)	0.0023 (5)	0.0034 (5)
O9	0.0278 (7)	0.0171 (7)	0.0435 (8)	0.0040 (5)	0.0079 (6)	0.0062 (6)
O10	0.0231 (6)	0.0186 (7)	0.0203 (6)	0.0041 (5)	−0.0020 (5)	0.0083 (5)
O11	0.0293 (7)	0.0380 (9)	0.0279 (7)	0.0037 (6)	−0.0054 (6)	0.0066 (6)
C1	0.0191 (8)	0.0156 (9)	0.0157 (8)	0.0049 (6)	0.0017 (6)	0.0041 (6)
C2	0.0167 (7)	0.0166 (9)	0.0148 (7)	0.0051 (6)	0.0034 (6)	0.0054 (6)
C3	0.0162 (8)	0.0156 (8)	0.0150 (7)	0.0027 (6)	−0.0006 (6)	0.0013 (6)
C4	0.0170 (8)	0.0148 (8)	0.0189 (8)	0.0043 (6)	0.0022 (6)	0.0022 (6)
C5	0.0183 (8)	0.0166 (9)	0.0164 (7)	0.0052 (6)	0.0010 (6)	0.0048 (6)
C6	0.0189 (8)	0.0167 (8)	0.0182 (8)	0.0039 (6)	0.0004 (6)	0.0000 (6)
C7	0.0286 (10)	0.0169 (9)	0.0206 (9)	0.0073 (7)	0.0045 (7)	0.0024 (7)
C8	0.0365 (11)	0.0227 (10)	0.0228 (9)	0.0043 (8)	−0.0018 (8)	−0.0014 (7)
C9	0.0154 (8)	0.0147 (8)	0.0176 (8)	0.0043 (6)	−0.0011 (6)	0.0012 (6)
C10	0.0244 (9)	0.0298 (10)	0.0189 (8)	0.0116 (7)	0.0008 (6)	0.0075 (7)
C11	0.0334 (10)	0.0234 (10)	0.0172 (8)	0.0073 (8)	0.0028 (7)	0.0017 (7)
C12	0.0504 (14)	0.0434 (14)	0.0206 (10)	−0.0062 (11)	−0.0034 (9)	−0.0024 (9)
C13	0.0243 (9)	0.0171 (9)	0.0224 (8)	0.0046 (7)	−0.0001 (7)	0.0048 (7)
C14	0.0350 (11)	0.0218 (11)	0.0614 (16)	0.0118 (9)	0.0152 (10)	0.0073 (10)
C15	0.0191 (8)	0.0225 (10)	0.0161 (8)	0.0054 (7)	0.0032 (6)	0.0084 (6)
C16	0.0263 (9)	0.0229 (9)	0.0238 (8)	0.0090 (7)	0.0049 (7)	0.0102 (7)
C17	0.0243 (9)	0.0309 (11)	0.0258 (9)	0.0124 (8)	0.0043 (7)	0.0139 (8)
C18	0.0228 (9)	0.0310 (11)	0.0175 (8)	0.0068 (7)	0.0035 (7)	0.0101 (7)
C19	0.0254 (9)	0.0233 (10)	0.0160 (8)	0.0065 (7)	0.0019 (7)	0.0041 (7)
C20	0.0240 (9)	0.0229 (10)	0.0180 (8)	0.0085 (7)	0.0014 (7)	0.0070 (7)
C21	0.0223 (9)	0.0400 (13)	0.0235 (9)	0.0075 (8)	0.0010 (7)	0.0120 (9)

### Geometric parameters (Å, °)

**Table:**

O1—C5	1.413 (2)	C7—C8	1.499 (3)
O1—C1	1.439 (2)	C8—H8A	0.9800
O2—C7	1.340 (2)	C8—H8B	0.9800
O2—C6	1.443 (2)	C8—H8C	0.9800
O3—C7	1.210 (3)	C9—C10	1.492 (2)
O4—C9	1.361 (2)	C10—H10A	0.9800
O4—C2	1.443 (2)	C10—H10B	0.9800
O5—C9	1.199 (2)	C10—H10C	0.9800
O6—C11	1.360 (2)	C11—C12	1.497 (3)
O6—C3	1.4389 (19)	C12—H12A	0.9800
O7—C11	1.197 (3)	C12—H12B	0.9800
O8—C13	1.356 (2)	C12—H12C	0.9800
O8—C4	1.431 (2)	C13—C14	1.489 (3)
O9—C13	1.199 (3)	C14—H14A	0.9800
O10—C15	1.381 (2)	C14—H14B	0.9800
O10—C5	1.404 (2)	C14—H14C	0.9800
O11—C21	1.212 (3)	C15—C16	1.391 (3)
C1—C6	1.513 (2)	C15—C20	1.403 (3)
C1—C2	1.534 (2)	C16—C17	1.380 (3)
C1—H1	1.0000	C16—H16	0.9500
C2—C3	1.521 (2)	C17—C18	1.400 (3)
C2—H2	1.0000	C17—H17	0.9500
C3—C4	1.520 (2)	C18—C19	1.396 (3)
C3—H3	1.0000	C18—C21	1.472 (3)
C4—C5	1.527 (2)	C19—C20	1.384 (3)
C4—H4	1.0000	C19—H19	0.9500
C5—H5	1.0000	C20—H20	0.9500
C6—H6A	0.9900	C21—H21	0.9500
C6—H6B	0.9900		
			
C5—O1—C1	111.07 (12)	H8B—C8—H8C	109.5
C7—O2—C6	116.65 (14)	O5—C9—O4	122.82 (15)
C9—O4—C2	117.18 (13)	O5—C9—C10	127.05 (16)
C11—O6—C3	117.74 (14)	O4—C9—C10	110.10 (14)
C13—O8—C4	117.05 (14)	C9—C10—H10A	109.5
C15—O10—C5	115.77 (13)	C9—C10—H10B	109.5
O1—C1—C6	106.88 (13)	H10A—C10—H10B	109.5
O1—C1—C2	107.27 (13)	C9—C10—H10C	109.5
C6—C1—C2	113.52 (14)	H10A—C10—H10C	109.5
O1—C1—H1	109.7	H10B—C10—H10C	109.5
C6—C1—H1	109.7	O7—C11—O6	124.08 (17)
C2—C1—H1	109.7	O7—C11—C12	126.49 (19)
O4—C2—C3	104.57 (13)	O6—C11—C12	109.41 (17)
O4—C2—C1	111.42 (13)	C11—C12—H12A	109.5
C3—C2—C1	110.19 (13)	C11—C12—H12B	109.5
O4—C2—H2	110.2	H12A—C12—H12B	109.5
C3—C2—H2	110.2	C11—C12—H12C	109.5
C1—C2—H2	110.2	H12A—C12—H12C	109.5
O6—C3—C2	107.91 (13)	H12B—C12—H12C	109.5
O6—C3—C4	108.09 (14)	O9—C13—O8	123.37 (18)
C2—C3—C4	111.16 (13)	O9—C13—C14	125.80 (19)
O6—C3—H3	109.9	O8—C13—C14	110.81 (17)
C2—C3—H3	109.9	C13—C14—H14A	109.5
C4—C3—H3	109.9	C13—C14—H14B	109.5
O8—C4—C3	108.55 (14)	H14A—C14—H14B	109.5
O8—C4—C5	107.52 (13)	C13—C14—H14C	109.5
C3—C4—C5	109.22 (14)	H14A—C14—H14C	109.5
O8—C4—H4	110.5	H14B—C14—H14C	109.5
C3—C4—H4	110.5	O10—C15—C16	116.66 (17)
C5—C4—H4	110.5	O10—C15—C20	122.13 (16)
O10—C5—O1	109.38 (13)	C16—C15—C20	121.21 (17)
O10—C5—C4	106.87 (14)	C17—C16—C15	119.31 (19)
O1—C5—C4	108.50 (13)	C17—C16—H16	120.3
O10—C5—H5	110.7	C15—C16—H16	120.3
O1—C5—H5	110.7	C16—C17—C18	120.59 (17)
C4—C5—H5	110.7	C16—C17—H17	119.7
O2—C6—C1	105.19 (14)	C18—C17—H17	119.7
O2—C6—H6A	110.7	C19—C18—C17	119.37 (17)
C1—C6—H6A	110.7	C19—C18—C21	121.21 (18)
O2—C6—H6B	110.7	C17—C18—C21	119.42 (18)
C1—C6—H6B	110.7	C20—C19—C18	120.91 (18)
H6A—C6—H6B	108.8	C20—C19—H19	119.5
O3—C7—O2	123.64 (17)	C18—C19—H19	119.5
O3—C7—C8	125.73 (18)	C19—C20—C15	118.61 (17)
O2—C7—C8	110.58 (16)	C19—C20—H20	120.7
C7—C8—H8A	109.5	C15—C20—H20	120.7
C7—C8—H8B	109.5	O11—C21—C18	124.9 (2)
H8A—C8—H8B	109.5	O11—C21—H21	117.5
C7—C8—H8C	109.5	C18—C21—H21	117.5
H8A—C8—H8C	109.5		
			
C5—O1—C1—C6	−170.55 (13)	C3—C4—C5—O1	58.77 (17)
C5—O1—C1—C2	67.38 (15)	C7—O2—C6—C1	−174.96 (14)
C9—O4—C2—C3	140.73 (14)	O1—C1—C6—O2	64.96 (16)
C9—O4—C2—C1	−100.26 (16)	C2—C1—C6—O2	−176.99 (13)
O1—C1—C2—O4	−173.01 (13)	C6—O2—C7—O3	−2.8 (3)
C6—C1—C2—O4	69.16 (17)	C6—O2—C7—C8	174.88 (15)
O1—C1—C2—C3	−57.40 (16)	C2—O4—C9—O5	9.6 (2)
C6—C1—C2—C3	−175.23 (13)	C2—O4—C9—C10	−168.33 (15)
C11—O6—C3—C2	117.13 (16)	C3—O6—C11—O7	2.1 (3)
C11—O6—C3—C4	−122.56 (16)	C3—O6—C11—C12	−176.77 (19)
O4—C2—C3—O6	−69.99 (15)	C4—O8—C13—O9	2.9 (3)
C1—C2—C3—O6	170.17 (13)	C4—O8—C13—C14	−178.48 (17)
O4—C2—C3—C4	171.65 (13)	C5—O10—C15—C16	−134.15 (17)
C1—C2—C3—C4	51.81 (18)	C5—O10—C15—C20	46.8 (2)
C13—O8—C4—C3	−110.11 (16)	O10—C15—C16—C17	−178.90 (15)
C13—O8—C4—C5	131.85 (15)	C20—C15—C16—C17	0.2 (3)
O6—C3—C4—O8	73.14 (16)	C15—C16—C17—C18	−0.2 (3)
C2—C3—C4—O8	−168.61 (13)	C16—C17—C18—C19	0.2 (3)
O6—C3—C4—C5	−169.91 (13)	C16—C17—C18—C21	−179.67 (17)
C2—C3—C4—C5	−51.66 (18)	C17—C18—C19—C20	−0.2 (3)
C15—O10—C5—O1	−89.74 (17)	C21—C18—C19—C20	179.63 (17)
C15—O10—C5—C4	152.99 (14)	C18—C19—C20—C15	0.2 (3)
C1—O1—C5—O10	175.14 (12)	O10—C15—C20—C19	178.82 (15)
C1—O1—C5—C4	−68.62 (16)	C16—C15—C20—C19	−0.2 (3)
O8—C4—C5—O10	−65.78 (16)	C19—C18—C21—O11	−1.9 (3)
C3—C4—C5—O10	176.62 (13)	C17—C18—C21—O11	177.97 (19)
O8—C4—C5—O1	176.37 (13)		

### Hydrogen-bond geometry (Å, °)

**Table:**

*D*—H···*A*	*D*—H	H···*A*	*D*···*A*	*D*—H···*A*
C1—H1···O5^i^	1.00	2.51	3.356 (2)	143
C3—H3···O5^i^	1.00	2.35	3.207 (2)	143
C6—H6A···O9^ii^	0.99	2.40	3.324 (2)	155
C8—H8C···O11^iii^	0.98	2.54	3.475 (3)	160

Symmetry codes: (i) *x*+1, *y*, *z*; (ii) *x*, *y*+1, *z*; (iii) *x*−1, *y*, *z*.
